# Berberine-taxifolin co-administration attenuates inflammatory response and intestinal barrier injury via nf-κB/NLRP3 suppression in colitis

**DOI:** 10.3389/fimmu.2025.1725084

**Published:** 2026-01-21

**Authors:** Ganggang Miao, De Zhang, Zhenghui Sui, Jianfei Leng, Yanxiang Deng, Xingwei Gu, Hongyong Cao

**Affiliations:** 1 Department of General Surgery, Nanjing First Hospital, Nanjing Medical University, Nanjing, Jiangsu, China; 2 Department of General Surgery, The People’s Hospital of Danyang, Affiliated Danyang Hospital of Nantong University, Danyang, Zhenjiang, China; 3 Department of Surgery, Universitätsklinikum Erlangen, Friedrich-Alexander Universität Erlangen-Nürnberg, Erlangen, Germany; 4 Department of General Surgery, The Hospital of Nanjing Qixia District, Nanjing, Jiangsu, China

**Keywords:** berberine, inflammatory bowel disease, inflammatory response, intestinal barrier damage, taxifolin

## Abstract

Inflammatory bowel disease (IBD) is pathologically characterized by dysregulated inflammation and compromised intestinal barrier integrity. While multi-component herbal formulations hold promise for IBD management, the combined potential of specific phytochemical combinations remains underexplored. This study investigates the cooperative therapeutic effects of Berberine and Taxifolin, two anti-inflammatory phytochemicals, in a murine colitis model. Multi-omics network pharmacology initially identified their shared anti-inflammatory and anti-apoptotic targets in IBD pathogenesis. Experimental validation demonstrated that combined treatment with berberine and taxifolin produced stronger protective effects against Dextran Sulfate Sodium (DSS)-induced colitis than either compound alone. Specifically, the combination significantly alleviated body weight loss and colon shortening, reduced macrophage infiltration and the expression of pro-inflammatory cytokines (IL-1β and TNF-α), and preserved intestinal barrier integrity by restoring tight junction proteins (occludin and ZO-1). In addition, the combined treatment attenuated caspase-3-mediated epithelial apoptosis. Molecular docking analysis suggested that berberine and taxifolin may interact with multiple inflammation-related targets, including NF-κB, NLRP3, PPARγ, and STAT3, providing a potential mechanistic basis for the observed effects. These findings establish Berberine-Taxifolin co-administration as a novel multi-target therapeutic strategy that concurrently addresses inflammatory dysregulation, barrier repair, and apoptosis control in IBD, and may provide a phytochemical blueprint for complex inflammatory disorders.

## Introduction

Inflammatory bowel disease (IBD), an idiopathic inflammatory condition affecting the ileum, rectum, and colon—including ulcerative colitis (UC) and Crohn’s disease (CD)—is clinically characterized by diarrhea, abdominal pain, and, in severe cases, bloody mucous stools. Incidence of IBD is sharply increasing in all age groups worldwide, placing a significant medical and economic burden on healthcare systems (1–3). Although the pathogenesis of UC is still unclear, studies have provided evidence that intestinal barrier damages, excessive inflammatory response, and oxidative stress are closely related to this disease (4–6). At present, amino salicylic acid, glucocorticoids, immunosuppressants, and other drugs are commonly used in the clinical treatment of UC, but these drugs have serious side effects such as gastrointestinal adverse effects (7), metabolic disorders (8), and immunosuppression (9–12). Notably, the complex pathogenesis and diverse clinical manifestations of colitis make single-agent conventional therapy inadequate for effectively alleviating acute symptoms while sustaining long-term efficacy. Therefore, it is highly crucial to discover new effective strategies for treating IBD.

Research has reported various protective effects of traditional Chinese herbal medicines in IBD, including the suppression of excessive inflammatory responses, inhibition of intestinal cell apoptosis, enhancement of intestinal barrier integrity to promote tissue repair (13, 14). However, the bioavailability of herbal medicines remains a significant limitation, and high doses may lead to severe adverse drug effects. Combination therapy, a hallmark of traditional Chinese medicine (TCM), has proven effective in enhancing drug bioavailability and therapeutic efficacy (15, 16). Despite evidence supporting the benefits of herbal combinations, effective strategies for using combined herbal therapies to treat IBD are currently lacking. Berberine is a quaternary ammonium alkaloid extracted from *Coptis chinensis*. It is one of the primary active antibacterial components of Coptis. Studies have demonstrated that Berberine alleviates the pathological symptoms of IBD by modulating gut microbiota, inhibiting intestinal cell apoptosis, reducing inflammation, and improving gut function by inhibiting NF-κB and NLRP3 signaling pathway, both of which play central roles in intestinal inflammation (17–19). NF-κB is a key transcription factor that regulates the expression of various inflammatory and immunoregulatory genes, cell cycle regulating genes, anti-apoptotic genes (20). NLRP3, a cytoplasmic inflammasome sensor, mediates the activation of caspase-1 and promotes the maturation and release of inflammatory cytokines such as IL-1β (21). Taxifolin, a flavonoid derived from the roots of *larch trees* in alpine regions, is a vital natural antioxidant with various pharmacological properties, including antitumor, antiviral, antibacterial, and anti-inflammatory effects (22–25). Some studies have shown that oral administration of Taxifolin has been shown to inhibit the NF-κB pathway to reduce pathological symptoms in Dextran Sulfate Sodium (DSS)-induced colitis mouse models by suppressing inflammatory cell infiltration and modulating gut microbiota (26–28). However, whether a combination of Berberine and Taxifolin can enhance therapeutic effects remains unclear.

Network pharmacology, based on systems biology, is a novel discipline that analyzes biological networks and identifies specific signaling nodes for multi-target drug design. By integrating systems biology with pharmacological principles, network pharmacology explores the relationships between biological networks and drug action networks, shifting from single-target discovery to comprehensive network analysis (29, 30). Molecular docking techniques further refine target selection based on network pharmacology findings (31).

In this study, we primarily investigated whether the combination of Berberine and Taxifolin enhances therapeutic efficacy in IBD and explored the underlying mechanisms.

## Materials and methods

### Prediction of drug targets for berberine and taxifolin and targets for colitis

The Simplified Molecular Input Line Entry System (SMILES) files of Berberine and Taxifolin were obtained from PubChem (https://pubchem.ncbi.nlm.nih.gov). Subsequently, several online databases were utilized to identify potential drug targets of Berberine and Taxifolin, including Traditional Chinese Medicine Systems Pharmacology Database and Analysis Platform (TCMSP) (https://tcmsp-e.com/tcmsp.php), Swiss Target Prediction database (http://swisstargetprediction.ch/), Encyclopedia of Traditional Chinese Medicine database (http://www.tcmip.cn/ETCM/index.php/Home/), and STITCH (http://stitch.embl.de/).

The molecular targets associated with colitis were identified through the Symptom Mapping database (http://www.symmap.org/), OMIM database (https://omim.org/), and Therapeutic Target Database (https://db.idrblab.net/ttd/). By intersecting the molecular targets of Berberine, Taxifolin, and colitis using a Venn diagram, a total of 37 molecular targets were obtained.

### Construction of the protein-protein interaction network, gene ontology and Kyoto encyclopedia of genes and genomes enrichment analyses

The PPI network analysis was conducted using the STRING database (https://cn.string-db.org/) to examine interactions among 37 compounds. The PPI network was subsequently analyzed, and hub genes were identified. Visual evaluation of the data was performed using Cytoscape 3.9.0 software. GO and Kyoto Encyclopedia of Genes and Genomes (KEGG) enrichment analyses were carried out using the ClusterProfiler tool in R version 3.18 (32–34).

### Experimental animals

Eight-week-old female C57BL/6J mice, weighing 20 ± 2 g, were obtained from Nanjing Medical University. All animal handling procedures were conducted in accordance with the regulations set forth by the Animal Protection and Use Committee of the Animal Ethics Committee of Nanjing Hospital affiliated to Nanjing Medical University and approved by the Institutional Animal Care and Use Committee (IACUC) of the Ethics Committee of The Affiliated Nanjing Hospital of Nanjing Medical University. Prior to the experiment, all animals were allowed a one-week period for environmental acclimation.

### Reagents, chemicals and cell cultures

DSS was obtained from Yeasen Biotechnology (60316ES60, Shanghai, China). Berberine was purchased from the MedChemExpress (HY-N0716, USA). Taxifolin was obtained from MedChemExpress (HY-N0136, USA). Caco-2 cells were cultured at 37°C in a humidified atmosphere with 5% CO_2_ in DMEM/F12 medium supplemented with 10% FBS and 1% penicillin–streptomycin. Targeted *in vitro* experiments were conducted by treating cells with TNF-α, berberine and taxifolin, as previously described (35, 36).

### DSS-induced models and the administration of berberine and/or taxifolin

All experimental animals were randomly and equally divided into five groups: Normal, DSS-induced colitis group, DSS-induced colitis + Berberine treated group, DSS-induced colitis + Taxifolin treated group, and DSS-induced colitis + Berberine + Taxifolin treated group. The colitis and drug treatment groups received drinking water containing 3% DSS for 5 days, while the normal group was given water without DSS. Berberine alone was administered at 20 mg/kg/d by oral gavage (37), and Taxifolin alone was orally administered at 100 mg/kg/d (28). And in the combination group, Berberine (10 mg/kg/d) + Taxifolin (50 mg/kg/d) was used. All drugs were administered at a gavage volume of 10 mL/kg (0.2 mL per 20 g mouse). The dosages were selected based on previously reported effective ranges in DSS-induced colitis models and adjusted according to body weight (28, 37). Following the establishment of DSS-induced colitis models, drug administration continued for an additional 9 days. Throughout the experiment, the Normal group received pure water, while the other groups had ad libitum access to a 3% (w/v) DSS solution. On the 14th day, mice were euthanized by intraperitoneal injection of pentobarbital sodium (150 mg/kg), and colon tissues were collected for further analysis. We conducted two independent animal experiments. The first batch included: Normal group (N = 10), DSS group (N = 14, with 4 deaths during the experiment), DSS + Berberin group (N = 10, no deaths), DSS + Taxifolin group (N = 10, no deaths), and DSS + Berberin + Taxifolin group (N = 10, no deaths), with this batch primarily utilized for body weight monitoring, immunohistochemistry, and immunofluorescence analyses. The second batch consisted of: Normal group (N = 8), DSS group (N = 11, with 3 deaths during the experiment), DSS + Berberin group (N = 8, no deaths), DSS + Taxifolin group (N = 8, no deaths), and DSS + Berberin + Taxifolin group (N = 8, no deaths), with these animals mainly employed for colon length measurement, Western blot (WB), and Real-time Quantitative Polymerase Chain Reaction (PCR) analyses.

### Body weight measurement and colitis severity evaluation

Mouse body weight was monitored throughout the modeling and drug administration process. Additionally, daily body weight changes were calculated and analyzed. The colon length and histological features of each mouse were evaluated and compared to those of control mice to assess the severity of colitis.

In all DSS-induced models, mice were monitored daily to record pathological features, including stool consistency, presence of blood in the stool, and body weight loss. For each parameter, a score from 0 to 4 was assigned, and the three subscores were then summed to generate the disease activity index (DAI) for each mouse (maximum score = 12), as previously described (38). Body weight loss was scored as follows: 0, no loss; 1, 1–5%; 2, 5–10%; 3, 10–20%; and 4, >20%. Stool consistency was scored as 0, normal; 1, loose stool; 2, watery diarrhea; 3, slimy diarrhea, little blood; and 4, severe watery diarrhea with blood. Blood stool was scored as 0, no blood; 2, presence of blood; and 4, gross bleeding.

### FITC–dextran permeability assay

Intestinal permeability was evaluated using a fluorescein isothiocyanate–dextran (FITC–dextran) assay. Briefly, mice were fasted for 4 h with free access to water and then orally gavaged with FITC–dextran (Sigma-Aldrich) dissolved in sterile PBS at a dose of 600 mg/kg body weight. Four hours after gavage, blood was collected to obtain serum. Serum samples were then measured for fluorescence intensity using a microplate reader. FITC–dextran concentrations were calculated from a standard curve generated using serial dilutions of FITC–dextran in mouse serum.

### Tissue processing and hematoxylin and eosin staining

Mice underwent perfusion with Phosphate-Buffered Saline (PBS) until blood clearance, followed by 4% paraformaldehyde infusion until tail stiffening. Colon tissue was promptly excised and immersed in 4% paraformaldehyde for external fixation. Subsequently, the colon tissue underwent dehydration, permeation, and embedding processes. Colon sections were cut to a thickness of 4μm. HE was performed according to the manufacturer’s protocol. Briefly, the process involved hematoxylin staining, differentiation, and bluing, followed by eosin staining. The sections were then dehydrated, cleared, and sealed. Finally, the stained sections were examined under an optical microscope and photographed.

### TUNEL staining

The TUNEL staining procedure was executed as follows: Initially, the fixed tissue sections were dewaxed, hydrated, and treated with proteinase K to expose the DNA ends. Subsequently, the sections were immersed in TUNEL reaction solution for incubation, allowing the fluorescently labeled dUTP to bind to the DNA break ends of apoptotic cells under the action of TdT enzyme. Finally, the sections were rinsed with PBS, sealed, and observed under a fluorescence microscope. The resulting fluorescent signal indicated the location of apoptotic cells.

### Immunofluorescence staining and laser confocal imaging

Following dewaxing and hydration of the tissue slices, we blocked them in 10% donkey serum/Phosphate-Buffered Saline with Tween-20 (PBST) for 1 hour at room temperature before incubating them with primary antibodies overnight at 4°C. The primary antibodies utilized in the assays were anti-claudin-1 (Proteintech, 28674-1-AP, 1:500), anti-MUC2 (Abcam, ab272692, 1:500), and anti-F4/80 (ThermoFisher, 14-4801-85, 1:300). Subsequently, the slices were rinsed three times with PBS, each for five minutes, before incubation with Alexa Fluor 488/555 secondary antibodies (1:1000 dilution, Invitrogen, United States) for one hour at room temperature. The nuclei were then stained with 4’,6-Diamidino-2-Phenylindole (DAPI) dye solution (YEASEN, Shanghai, China) for 30 minutes. Images were obtained using a laser confocal microscope and quantitative analysis by ImageJ software.

### RNA extraction and quantitative real-time PCR

Mouse colonic tissues underwent quantitative real-time PCR analysis following RNA extraction. Total RNA was isolated from colonic tissue specimens using RNAiso Plus reagent (Takara, #9108Q) following standardized protocols. Reverse transcription was performed with HiScript II Q RT SuperMix for qPCR (+gDNA wiper; Vazyme, Nanjing, China) to generate complementary DNA (cDNA). Subsequent qPCR amplification was conducted using ChamQ SYBR qPCR Master Mix (Vazyme, Nanjing, China) for mRNA quantification. Expression levels were normalized against the housekeeping gene β-actin, with fold-change calculations performed through the comparative threshold cycle (2−ΔΔCt) method. The primers used were listed: IL-1β(F: GCAACTGTTCCTGAACTCAACT, R: ATCTTTTGGGGTCCGTCAACT); IL-6 (F: CCAAGAGGTGAGTGCTTCCC, R: CTGTTGTTCAGACTCTCTCCCT); TNF-α (F: GACGTGGAACTGGCAGAAGAG, R: TTGGTGGTTTGTGAGTGTGAG).

### WB analysis

Following scarification, fresh colon tissue was collected. Protein extraction was performed by homogenizing colon tissue in ice-cold RIPA lysis buffer (Beyotime Biotechnology, China) supplemented with protease and phosphatase inhibitors with ultrasonic grinding. The lysates were incubated on ice for 20–30 minutes to ensure complete cell lysis, followed by centrifugation at 12,000–14,000 × g for 15 minutes at 4°C. The concentration of lysis protein was measured using the Bicinchoninic Acid (BCA) protein quantification kit (Key GENE BioTech, China). Protein samples were analyzed using 10% Sodium Dodecyl Sulfate–Polyacrylamide Gel Electrophoresis (SDS-PAGE) and transferred to a Polyvinylidene Fluoride (PVDF) Membrane (Millipore, USA). After blocking with 5% Bovine Serum Albumin (BSA) in Tris-Buffered Saline with Tween-20 (TBST), primary antibodies were incubated, followed by secondary antibodies conjugated with horseradish peroxidase (Proteintech, China). Chemiluminescent signals were detected using ECL kits (Tanon, China) and an exposure instrument (Tanon, China). The primary antibodies used in this experiment included: anti-caspase3 p17/p19 (Proteintech, 19677-1-AP, 1:1000), anti-Bax (Proteintech, 50599-2-Ig, 1:1000), anti-Bcl-2 (Proteintech, 26593-1-AP, 1:1000), anti-occludin (Proteintech, 27260-1-AP, 1:10000), anti-ZO-1 (Abcam, ab307799, 1:1000), anti-IL-1β (Proteintech, 16806-1-AP, 1:1000), anti-iNOS (Proteintech, 22226-1-AP, 1:1000), anti-TNFα (Abcam, ab183218, 1:1000), anti-IL-6 (Abcam, ab7737, 1:1000), anti-NLRP3 (Abcam, ab263899, 1:1000), anti-p-NF-κB (CST, #3033, 1:1000), and β-Actin (Proteintech, 66009-1-Ig, 1:2000).

### Molecular docking analysis

The three-dimensional protein structures of core targets were obtained from the Protein Data Bank (PDB) database (https://www.rcsb.org/), including NF-κB1 (PDB ID: 1U36), PPARγ (PDB ID: 8B8W), NLRP3 (PDB ID: 3QF2), and STAT3 (PDB ID: 3CWG). The chemical structures of Berberine (PubChem CID: 2353) and Taxifolin (PubChem CID: 439533) were retrieved from the PubChem database (https://pubchem.ncbi.nlm.nih.gov). Prior to molecular docking, protein structures were preprocessed using AutoDock, including the removal of water molecules and co-crystallized ligands, the addition of polar hydrogen atoms, and the assignment of partial charges. AutoDock software was then employed to perform molecular docking between the target proteins and the compounds Berberine and Taxifolin. Visualization of the docking results was accomplished using Discovery Studio software 2024.

### Statistical analysis

All data were presented as the mean ± SD. Statistical analysis and graphical representation were performed using GraphPad Prism 8.0. The significance of differences between groups was determined through one-way ANOVA followed by Dunnett’s *post hoc* multiple-comparison test. All datasets met the assumptions for parametric analysis, including approximate normality and homogeneity of variance. *p* value < 0.05 was considered statistically significant.

## Results

### Network analysis of berberine and taxifolin against colitis

**Figure 1:**
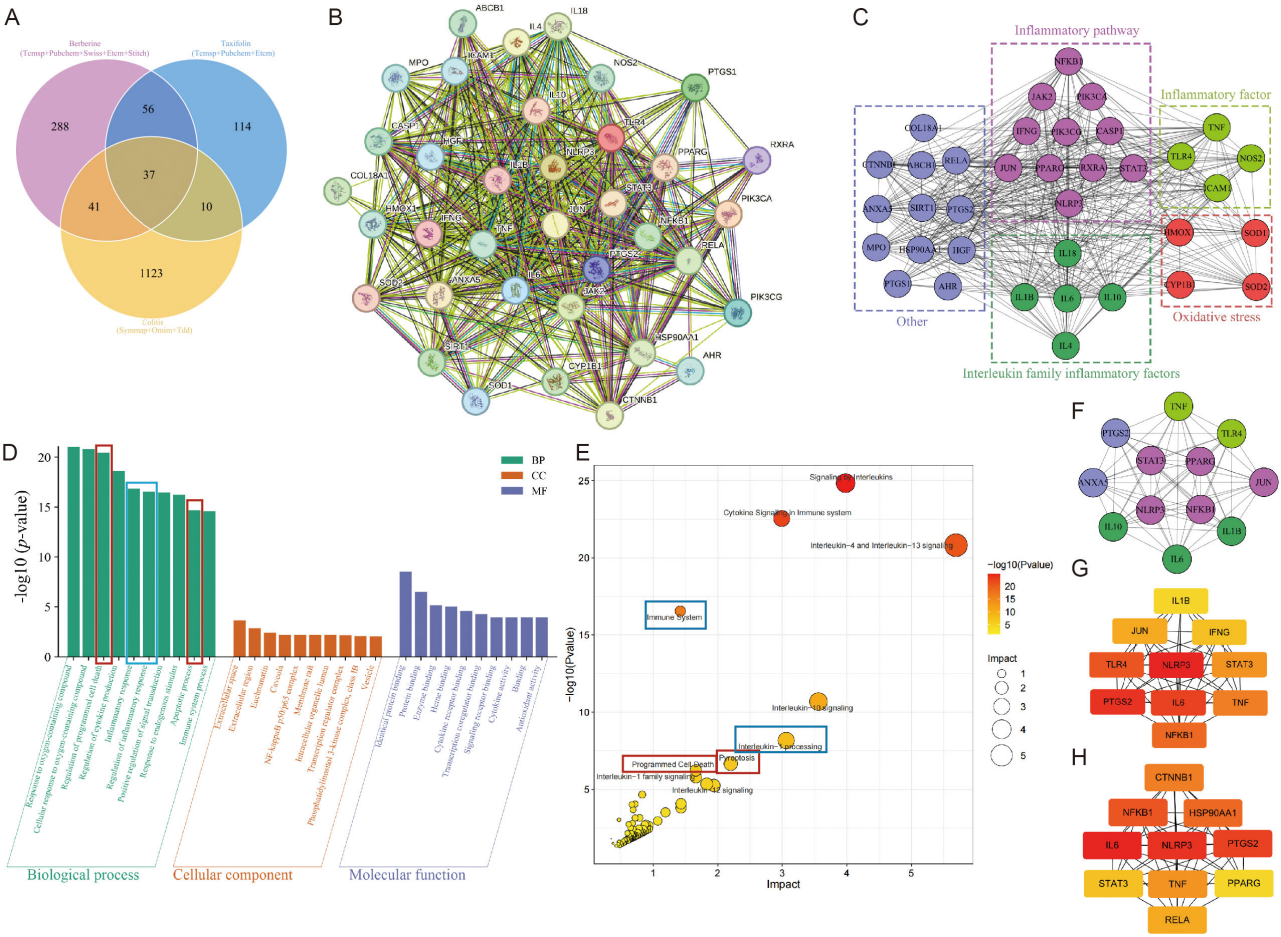
Prediction of intersection targets shared by colitis and drugs (Berberine and Taxifolin) and biological function analysis. **(A)** The molecular targets of Berberine and Taxifolin and the molecular targets of colitis were searched through online databases. The Venn diagram was used to intersect the molecular targets of Berberine, Taxifolin and colitis. **(B)** The PPI was used to perform interaction analysis on 37 molecules. **(C)** The 37 molecular targets were visualized and clustered using Cytoscape software. **(D)** GO enrichment analysis was performed on 37 molecules. **(E)** KEGG enrichment analysis was performed on 37 molecules. **(F)** Re-clustering by MCODE (degree>5) in Cytoscape software. **(G)** The top 10 molecular targets among 37 molecules were screened using the EPC statistical method in Cytoscape software. **(H)** The top 10 molecular targets among 37 molecules were screened using the MCC statistical method in Cytoscape software.

We first conducted network pharmacology analysis to preliminarily investigate whether the combination of Berberine and Taxifolin has the potential to treat colitis. The 422 molecular targets of Berberine were obtained from Temsp, Pubchem, Swiss, Etcm, and Stitch online database, the 217 molecular targets of Taxifolin were obtained from Tcmsp, Pubchem, and Etcm online database, and 1211 molecular targets of colitis were obtained from Symmap, Omim, and Tdd online database. The VENN analysis showed that the intersection of Berberine-related, Taxifolin-related, and colitis-related molecular targets yielded 37 common genes (**Figure 1A**), and moreover PPI analysis showed that the 37 common genes had strong interaction relationships (**Figure 1B**). Thus, we then conducted cluster analysis on the common genes to explore which pathological processes were mainly affected by the combination of Berberine and Taxifolin. As shown in **Figure 1C**, the 37 common genes were visually clustered, and they were predominantly associated with inflammation pathway, interleukin family inflammatory factors, and oxidative stress. In the GOBP, GOCC, and GOMF analysis, we found that the combination of Berberine and Taxifolin significantly exerted a potential role in regulation of programmed cell death, apoptotic process, and inflammatory response (**Figure 1D**). Besides, the KEGG analysis results showed that the combination of Berberine and Taxifolin regulated immune system, interleukin-1 processing, pyroptosis, and programmed cell death, which indicated that the combination of Berberine and Taxifolin could affect the inflammatory response and the apoptosis in colitis injury (**Figure 1E**). In addition, we conducted MCODE analysis to confirm the hub gene in the yielded 37 common genes (degree > 5) (**Figure 1F**) and we also checked the results by using EPC (**Figure 1G**) and MCC (**Figure 1H**) analysis, and results showed that NLRP3, STAT3, NF-κB, and PPARγ are the hub genes. Thus, the combination of Berberine and Taxifolin might interact with these core proteins to regulate the inflammatory response and the apoptosis in colitis injury.

### The combination of berberine and taxifolin enhanced therapeutic effects on colitis injury

**Figure 2:**
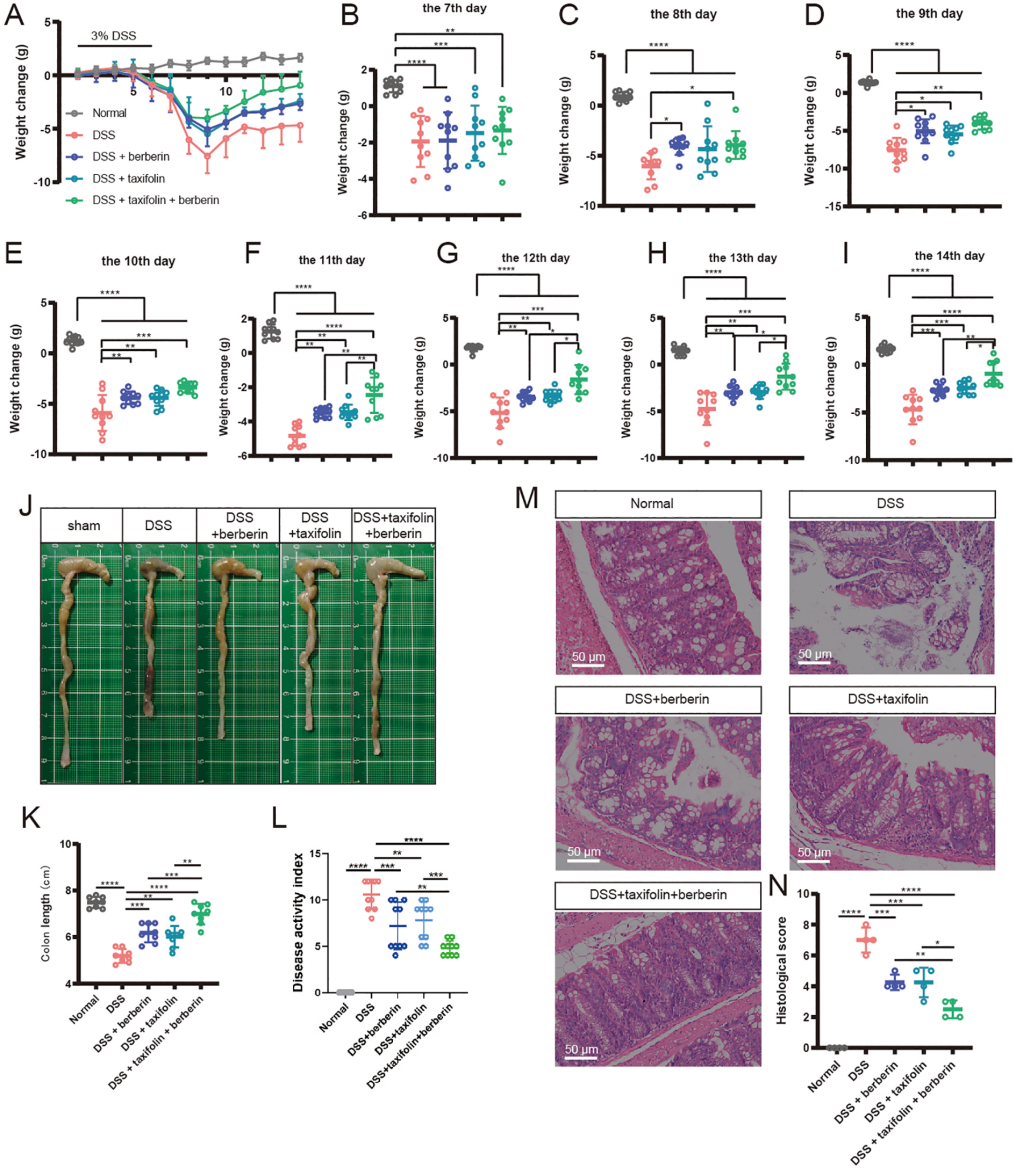
Berberine and Taxifolin combined administration increased the protective effect against colitis in mice. **(A)** Body weight changes of mice after modeling and drug administration. **(B–I)** Body weight changes of mice at modeling and drug administration 7^th^ day to 14^th^ day. **(J, K)** The colon length changes in different treatment groups. **(L)** Disease activity index in different treatment groups. **(M, N)** The H&E staining and Histological scores in different treatment groups. *p <0.05; **p <0.01; ***p <0.001, ****p <0.0001.

Next, we explored whether the combination of Berberine and Taxifolin could enhance the protective action. Using a mouse model, the *in vivo* intestine-protecting effects of Berberine alone (20mg/kg body weight), Taxifolin alone (100mg/kg body weight), and Berberine-Taxifolin combination (10mg/kg Berberine + 50mg/kg Taxifolin body weight) on 3% DSS-induced colitis were compared. First, we detected the weight of mice from the 7^th^ day to the 14^th^ day, and results showed that the administration with Berberine alone or Taxifolin alone significantly inhibited the reduction of weight, and Berberine-Taxifolin combination also significantly inhibited the reduction of weight and enhanced the protective action from 11^th^ day to 14^th^ day. Then, we detected the colon length of mice from different groups on the 14th day (**Figures 2A–I**). We found that compared to the Berberine alone or Taxifolin alone, the Berberine-Taxifolin combination significantly inhibited the reduction of the colon length of DSS-induced colitis mice (**Figures 2J, K**). In addition, disease activity index scores were significantly reduced by Berberine or Taxifolin treatment, with the Berberine–Taxifolin combination showing a more pronounced protective effect (**Figure 2L**). Moreover, histopathological analysis results from HE staining showed that the administration with Berberine alone or Taxifolin alone significantly decreased inflammatory cell infiltration and improved the integrity of mucosal structures, and Berberine-Taxifolin combination exerted greater effects (**Figures 2M, N**). Accordingly, Berberine and Taxifolin could work jointly to alleviate DSS-induced colitis injury.

### Combination of berberine and taxifolin enhanced intestinal barrier integrity

**Figure 3:**
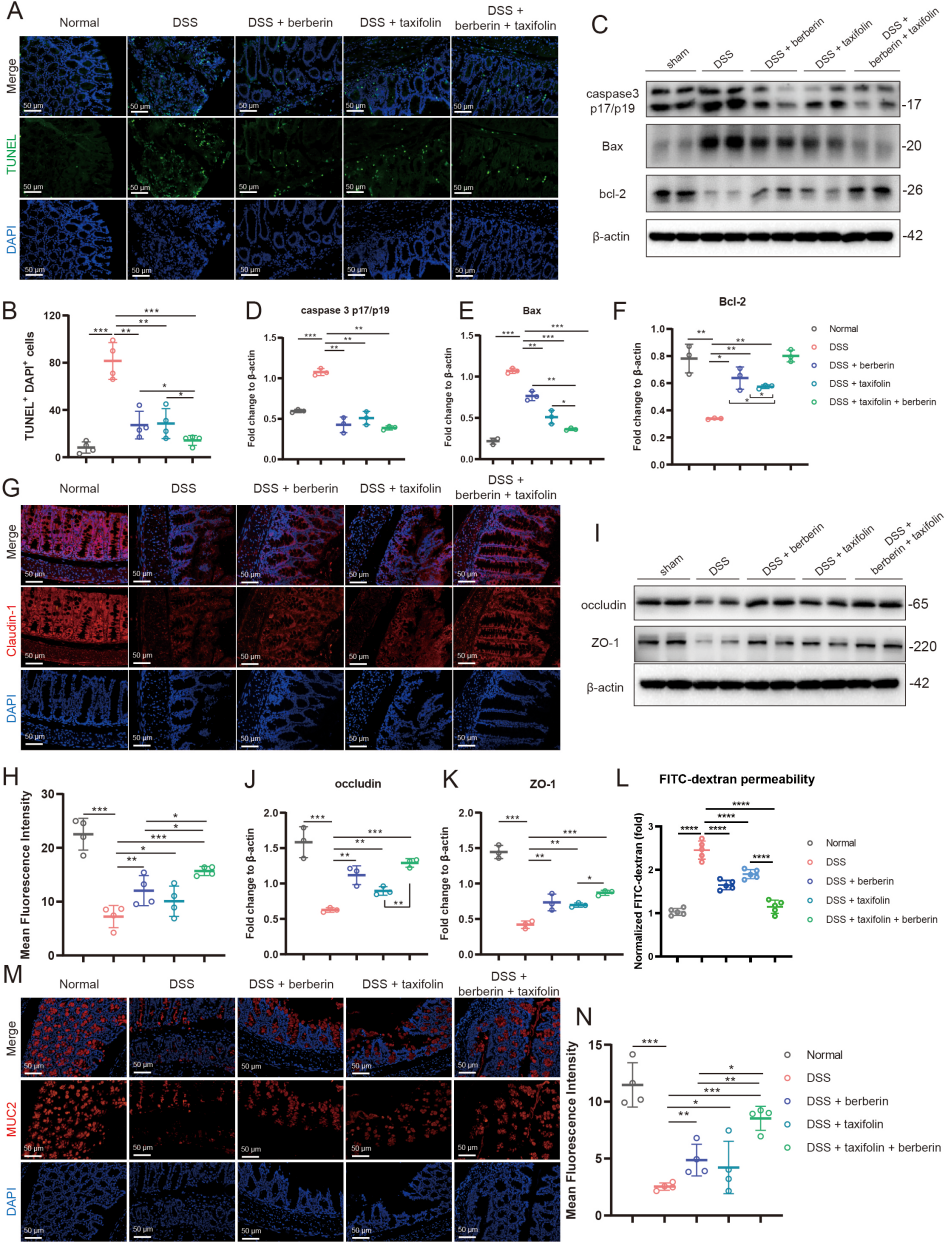
Berberine and Taxifolin combined administration inhibited colon tissue cell apoptosis and improved colon mucosal structure and permeability. **(A, B)** TUNEL staining analysis the apoptotic cell proportion changes in each group. **(C–F)** The expression changes of apoptotic related protein including caspase3 p17/p19, Bax and Bcl-2 in each group by western blotting. **(G, H)** Representative immunofluorescence imaging and statistical analysis of occludin in each group. **(I–K)** The expression changes of occludin and ZO-1 in each group by western blotting. **(L)** FITC–dextran permeability assay showing changes of intestinal barrier function. **(M, N)** Representative immunofluorescence imaging and statistical analysis of MUC2 in each group. *p <0.05; **p <0.01; ***p <0.001, ****p <0.0001.

Intestinal barrier damage is a key pathological feature of IBD. This study investigated whether the combination of Berberine and Taxifolin could improve this pathology. First, apoptosis in intestinal tissues was evaluated using TUNEL staining. Results showed that DSS treatment caused significant apoptosis in intestinal tissues, while both Berberine and Taxifolin alone markedly reduced apoptosis. Notably, their combination further inhibited intestinal apoptosis (**Figures 3A, B**). Next, we analyzed apoptosis-related proteins. Both Berberine and Taxifolin individually decreased the expression of pro-apoptotic proteins caspase-3 p17/p19 and Bax, while increasing the expression of the anti-apoptotic protein Bcl-2. Their combination further enhanced these effects (**Figures 3C–F**). These findings suggested that the combination of Berberine and Taxifolin significantly enhanced the anti-apoptotic effects in intestinal tissues. We also assessed the expression of tight junction proteins in intestinal tissues. Immunohistochemical staining revealed that Berberine and Taxifolin alone increased claudin-1 expression, and their combination led to an even greater increase (**Figures 3G, H**). Western blotting showed that the expression of occludin and ZO-1, reduced in colitis, was significantly restored by either compound alone or further increased by their combination (**Figures 3I–K**). Consistently, the FITC–dextran permeability assay showed that DSS-induced intestinal hyperpermeability was significantly alleviated by Berberine or Taxifolin treatment, with the combination group exhibiting a further reduction in serum FITC–dextran levels (**Figure 3L**). Additionally, MUC2, a marker of intestinal structural integrity, was significantly reduced in DSS-induced colitis but was restored by Berberine and Taxifolin, with the combination showing an enhanced effect (**Figures 3M, N**). Together, these results demonstrated that the combination of Berberine and Taxifolin further improved intestinal barrier integrity.

### Combination of berberine and taxifolin inhibited excessive inflammation

**Figure 4:**
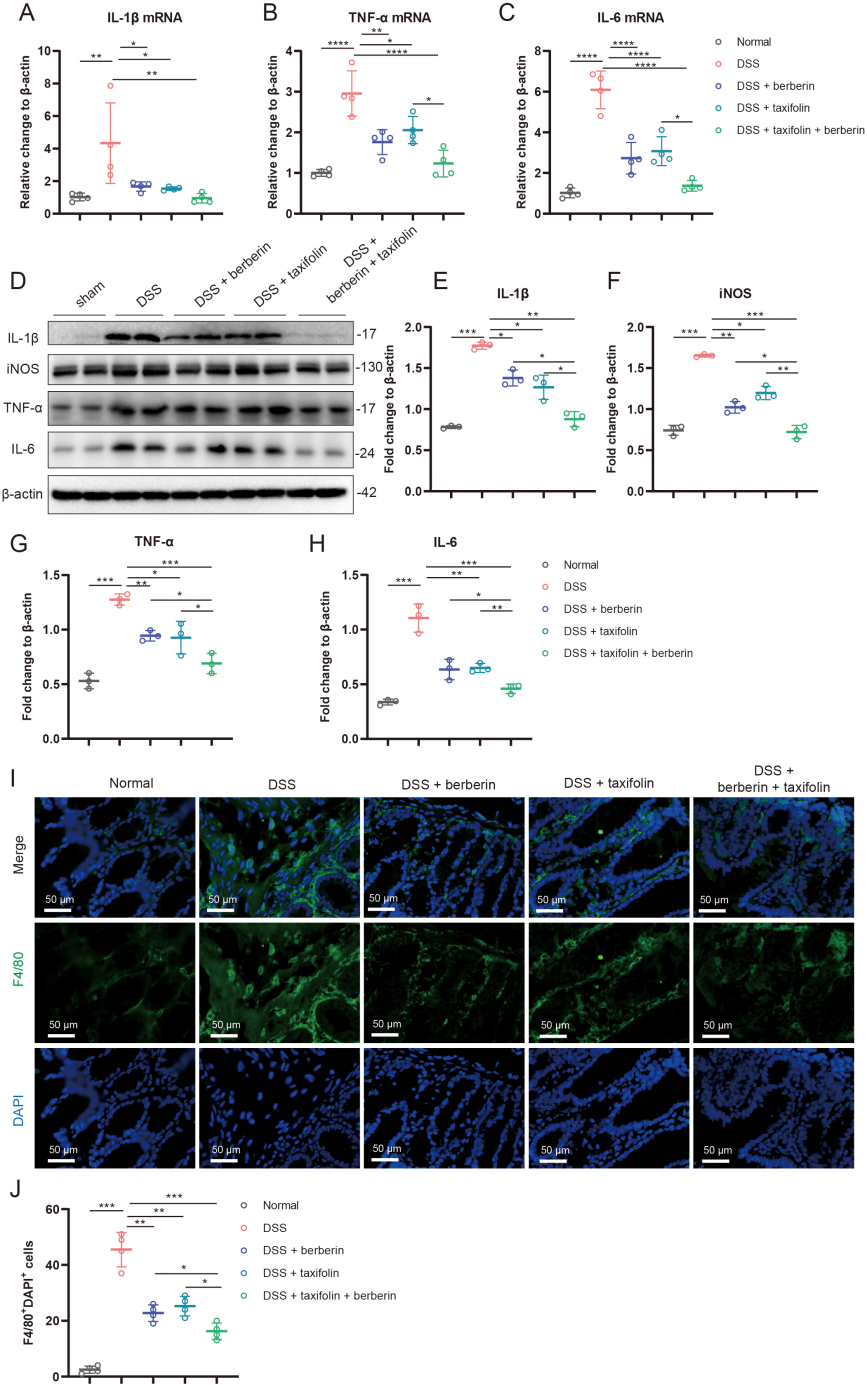
Berberine and Taxifolin combined administration ameliorated colon tissue inflammation and macrophage infiltration. **(A–C)** The mRNA expression changes of pro-inflammatory cytokines including IL-1β, iNOS, and TNF-α and IL-6 in each group by qRT-PCR. **(D–H)** The expression changes of pro-inflammatory cytokines including IL-1β, iNOS, and TNF-α and IL-6 in each group by western blotting. **(I, J)** Representative immunofluorescence imaging and statistical analysis of F4/80 in each group. *p <0.05; **p <0.01; ***p <0.001, ****p <0.0001.

Excessive inflammation, characterized by macrophage infiltration and the release of pro-inflammatory cytokines, exacerbates intestinal barrier damage and inflammation. This study evaluated whether the combination of Berberine and Taxifolin could mitigate such excessive inflammation. First, we quantitatively analyzed the mRNA expression levels of inflammatory cytokines in colonic tissues across experimental groups using real-time PCR. The results demonstrated that both Berberine and Taxifolin monotherapy significantly attenuated DSS-induced upregulation of IL-1β, TNF-α, and IL-6, while their combination therapy achieved further suppression of these pro-inflammatory cytokine expression levels (**Figures 4A–C**). Western blot results revealed that both compounds individually reduced the expression of pro-inflammatory cytokines IL-1β, iNOS, TNF-α, and IL-6, and their combination achieved further reductions (**Figures 4D–H**). Immunohistochemical staining of F4/80, a macrophage marker, showed that DSS induced significant macrophage infiltration in the colon. Both Berberine and Taxifolin alone reduced the number of F4/80+ macrophages, with the combination further decreasing their presence in the colonic tissue (**Figures 4I, J**). These findings indicate that the combination of Berberine and Taxifolin effectively suppresses excessive intestinal inflammation.

### Berberine and taxifolin interact with NF-κB1, NLRP3, PPARγ, and STAT3

**Figure 5:**
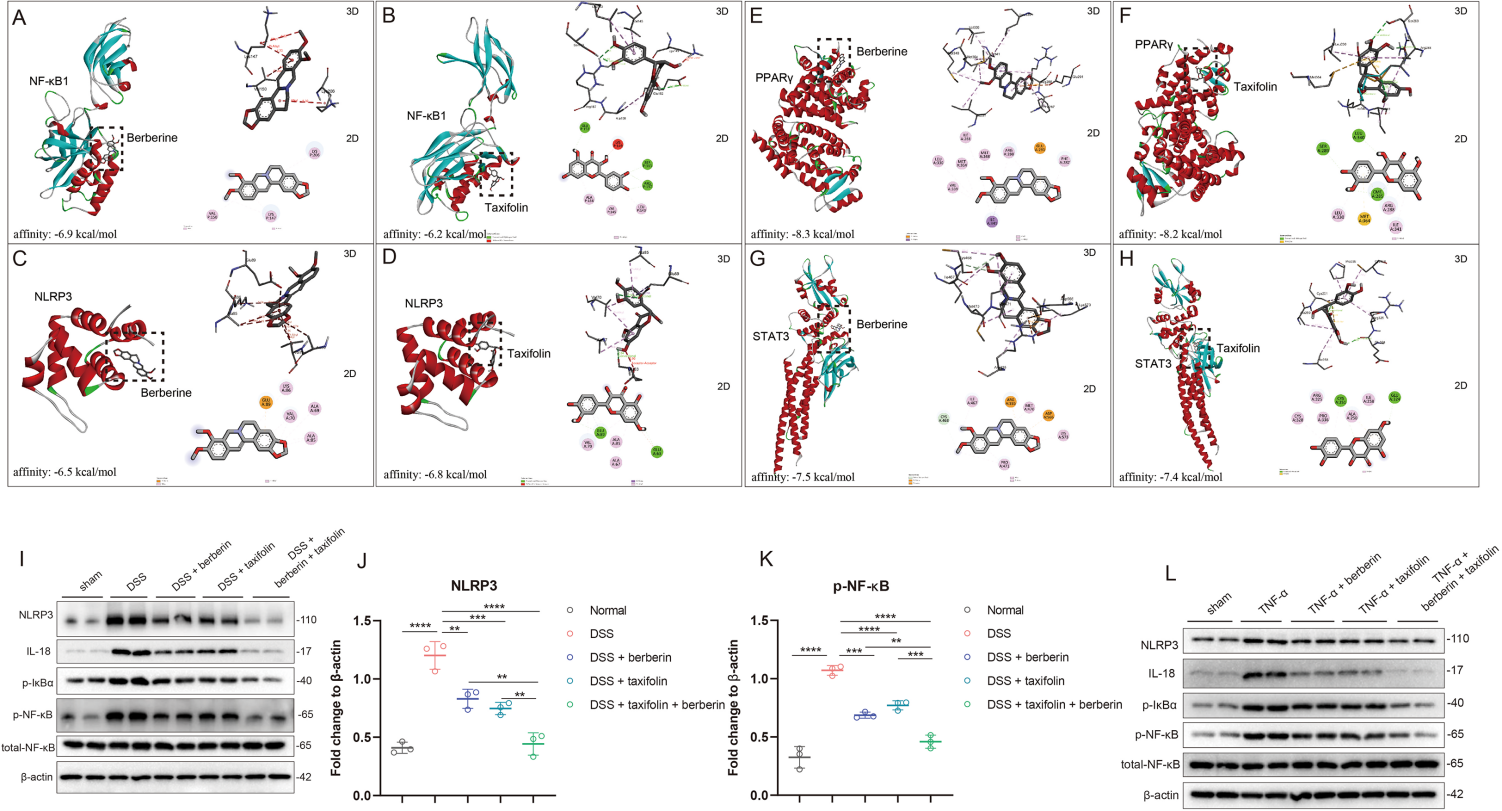
Berberine and Taxifolin could bind with key molecular targets screened based on network pharmacology by molecular docking. **(A, B)** Molecular docking between Berberine or Taxifolin and NF-κB1 and Images showing specific binding sites and interaction patterns. **(C, D)** Molecular docking between Berberine or Taxifolin and NLRP3 and Images showing specific binding sites and interaction patterns. **(E, F)** Molecular docking between Berberine or Taxifolin and PPARγ and Images showing specific binding sites and interaction patterns. **(G, H)** Molecular docking between Berberine or Taxifolin and STAT3 and Images showing specific binding sites and interaction patterns. **(I–K)** Western blot analysis of NLRP3 and NF-κB signaling–related proteins in each group. **(L)** Western blot analysis of NLRP3 and NF-κB signaling–related proteins *in vitro*. **p <0.01; ***p <0.001, ****p <0.0001.

Previous findings suggested that Berberine and Taxifolin might jointly target key molecules such as NF-κB1, NLRP3, PPARγ, and STAT3 to exert their anti-colitis effects. To investigate this hypothesis, we employed molecular docking to explore potential interactions between these compounds and the target proteins. Docking results showed that Berberine primarily interacted with NF-κB1 through residues Lys206, Lys147, and Val150, forming pi-alkyl interactions (**Figure 5A**); Taxifolin, in contrast, interacted with Leu143, Val145, and Ala156 residues of NF-κB1, also forming pi-alkyl interactions (**Figure 5B**). Similarly, both compounds could interact with NLRP3, Berberine targeted Lys86, Ala85, and Val70 residues of NLRP3 to form pi-alkyl interactions (**Figure 5C**), and Taxifolin targeted Val70, Ala57, and Ala85 residues of NLRP3 to form pi-alkyl interactions when targeted Glu89 and Glu63 to form conventional hydrogen bond (**Figure 5D**). Both Berberine and Taxifolin also could interact with STAT3 and PPARγ by forming pi-alkyl interactions and conventional hydrogen bond (**Figures 5E–H**). Furthermore, we performed western blot analysis to examine the expression levels of NLRP3 and key components of the NF-κB signaling pathway across different experimental groups. The results revealed that both Berberine and Taxifolin monotherapy significantly suppressed DSS-induced activation of the NLRP3 inflammasome and the NF-κB pathway, as evidenced by reduced expression of NLRP3, p-NF-κB (p-p65), p-IκBα, and IL-18. Notably, the combined administration of Berberine and Taxifolin resulted in a further significant reduction in the expression levels of these proteins (**Figures 5I–K**). Moreover, these regulatory effects on the NLRP3 inflammasome and NF-κB signaling pathway were further validated *in vitro* using a Caco-2 cell model (**Figure 5L**). Collectively, these findings suggested that Berberine and Taxifolin might exert their protective effects in colitis by interacting with NF-κB1, NLRP3, PPARγ, and STAT3, which could underpin their combined therapeutic mechanism.

## Discussion

IBD, also known as CD or UC, is characterized by impaired intestinal barrier and ongoing inflammation (39). The main classes of drugs for IBD include aminosalicylates (e.g., sulfasalazine) as first-line therapy, corticosteroids, immunosuppressives (e.g., azathioprine), and anti-TNF-α antibodies as biological agents (40, 41), but it remains the case that about 20% of ulcerative colitis and 60–75% for Crohn’s disease patients who don’t respond to medication require surgical intervention (39). Therefore, the number of ideal therapeutic drugs and treatment strategies remains limited for IBD. In recent years, the role of combination use of Chinese herbal medicine compounds in the prevention and treatment of IBD has gained significant attention. Berberine, an isoquinoline alkaloid isolated from the Chinese herb *Coptis chinensis* and other Berberis plants, has a wide range of pharmacological properties (42). Berberine exhibits therapeutic potential across various diseases, including cancer, digestive, metabolic, cardiovascular, and neurological disorders, by inhibiting pathogens, protecting intestinal and liver health, enhancing chemoradiotherapy efficacy, regulating metabolism, improving cardiovascular function, and exerting neuroprotective effects (43, 44). Taxifolin, a flavonoid compound originally isolated from Douglas fir bark and found in foods like onions and olive oil, is known for its antioxidant, anti-inflammatory, and antimicrobial properties, making it a subject of interest for nutritionists and medicinal chemists (22, 45). In this study, our findings reveal that Berberine and Taxifolin combined administration has an anti-IBD effect in DSS induced IBD model mice by inhibiting the apoptosis and inflammation.

Presently, unraveling the underlying mechanisms of Traditional Chinese Medicine (TCM) through modern medical approaches remains a significant challenge in the quest for its modernization (46). Network pharmacology, integrating systems biology and bioinformatics, offers a holistic strategy that has gained extensive application in TCM research, thereby presenting a novel pathway for exploring TCM mechanisms (47). In our study, the 422 molecular targets of Berberine, the 217 molecular targets of Taxifolin and 1211 molecular targets of colitis were obtained from online database. Meanwhile, the VENN analysis showed that the intersection of Berberine-related, Taxifolin-related, and colitis-related molecular targets yielded 37 common genes, which had been visually clustered for inflammation pathway, interleukin family inflammatory factors, and oxidative stress by PPI analysis. Notably, GO enrichment analysis indicated combination of Berberine and Taxifolin significantly exerted a potential role in regulation of programmed cell death, apoptotic process, and inflammatory response. And KEGG analysis revealed that Berberine and Taxifolin regulate immune responses, IL-1 processing, pyroptosis, and apoptosis, suggesting their potential to modulate inflammation and cell death in colitis. Based on MCODE analysis, EPC analysis and MCC analysis, NLRP3, STAT3, NF-κB, and PPARγ are found as the hub genes. In summary, we found the combination of Berberine and Taxifolin may interact with core proteins to regulate inflammation and apoptosis in colitis, which provide us a potential target to deeply study.

DSS-induced colitis model, a widely used animal model for studying IBD, has pathological and immunological characteristics similar to those of human IBD (48). In this study, the DSS-induced model was used to evaluate the curative effect of Berberine and Taxifolin combined administration on colitis. Body weight loss is the major characteristic of colitis (49), so we first access the body weight changes in our study. The results indicated that Berberine and Taxifolin combined administration markedly alleviated the mice body weight changes from the 8^th^ day to 14^th^ day. Interestingly, we also found the length and histopathology of mice colon were significantly improved after Berberine and Taxifolin combined administration. Although Berberine or Taxifolin can reduce damage when used alone, they are not as effective as when used together. Previous studies have shown that Berberine used alone has effects on weight loss and colon pathological progression in colitis (50), but there is little research on the therapeutic effect of Taxifolin on colitis (51). Therefore, we first conducted the combination of Berberine and Taxifolin administration exerts the unexpected effects in colitis, which may fill the gap of poor effect of current drugs in treating colitis.

Numerous investigations have confirmed intestinal epithelial barrier dysfunction and apoptosis results increase in intestinal permeability in IBD (52). Moreover, IBD progression involves dysregulation of tight junction proteins, loss of the mucus layer, and elevated epithelial cell death (53). Notably, in our study, we found Berberine and Taxifolin combined administration significantly relieved the apoptosis indicators including TUNEL staining and the increased expression of caspase3 p17/p19, Bax. Meanwhile, the anti-apoptotic protein Bcl-2 expression was increased after Berberine and Taxifolin combined administration. Similarly, the Berberine and Taxifolin combined administration provide greater protection than either alone. Our results also showed that the tight junction proteins occludin and ZO-1 levels were improved by combined administration. And the MUC2, a key indicators of intestinal barrier function, was dramatically increased after the combined treatment. These results confirmed the potential targets of network pharmacology analysis, which help us develop the combination of these two drugs as a potential treatment for IBD. Currently, there are few reports that the Berberine and Taxifolin alone or in combination can upregulate tight junctions, except for one article reporting the hypoglycemic effect of Berberine may be related to its effect on intestinal tight junctions and protection of the mucosal barrier (54). Our study further expands the function of the two drugs to protect tight junctions, which is crucial for the treatment of IBD, a disease characterized by loss of tight junctions.

Inflammatory cell infiltration and subsequent inflammatory response after intestinal epithelial tight junction destruction play an important role in the progression of IBD (55–57). The destruction of tight junctions promotes the infiltration of inflammatory cells such as F4/80-positive macrophages, amplifying the inflammatory response. We also found the number of F4/80 positive macrophages increased in the mice colon, and this phenomenon was reversed by Berberine and Taxifolin alone or in combination. Meanwhile, Berberine and Taxifolin alone or in combination also reduced the associated inflammatory response, which including the expression of IL-1β, iNOS, TNFα and IL-6. Previous studies have shown that Berberine can indeed inhibit oxidative stress and inflammation by inducing glucose metabolism by activating AMPK signaling (57), but the combination of Berberine and Taxifolin have a better anti-inflammatory effects.

In addition, we further explored the precise mechanism involved of the Berberine and Taxifolin exert protective effects in colitis by molecular docking. Unexpectedly, we found Berberine and/or Taxifolin could bind together some hub gene targets of our previous bioinformatics analysis results, which including NF-κB1, NLRP3, PPARγ and STAT3. Among these potential binding molecular, the NF-κB1 and STAT3 have more stronger bonding ability with Berberine and Taxifolin. This gives us sufficient confidence to develop drug combination into an important means of treating colitis. Although we have only evaluated the therapeutic effect of the two drugs in combination on colitis in terms of intestinal epithelial cell mucosal barrier and inflammation, we found that the combined effect of these two Chinese herbal compounds is indeed better than using them alone. Our research still has certain limitations. First, although molecular docking offers theoretical insights, the absence of experimental validation (e.g., co-IP, SPR, or mutagenesis assays) weakens the robustness of the proposed mechanistic interpretations. Second, the absence of functional validation using pathway-specific inhibition, knockout mouse models, or large-scale omics analyses limits the ability to establish definitive causal relationships.

In conclusion, our study showed that the combination of Berberine and Taxifolin exerted a protective effect in colitis mice, and its therapeutic mechanisms may be related to regulating the intestinal epithelial cell mucosal barrier permeability and inflammatory infiltration. Meanwhile, this study further determined that the Berberine combined with Taxifolin administration may exert the above protective effects by targeting NF-κB1 and STAT3 signaling. Our study highlights that the combination of Berberine and Taxifolin exerts an enhanced protective effect against colitis, which may open up new ideas for the treatment of colitis.

## Data Availability

The datasets presented in this study can be found in online repositories. The names of the repository/repositories and accession number(s) can be found in the article/supplementary material.
